# HIV Cell-to-Cell Spread Results in Earlier Onset of Viral Gene Expression by Multiple Infections per Cell

**DOI:** 10.1371/journal.ppat.1005964

**Published:** 2016-11-03

**Authors:** Mikaël Boullé, Thorsten G. Müller, Sabrina Dähling, Yashica Ganga, Laurelle Jackson, Deeqa Mahamed, Lance Oom, Gila Lustig, Richard A. Neher, Alex Sigal

**Affiliations:** 1 KwaZulu-Natal Research Institute for TB-HIV (K-RITH), Durban, South Africa; 2 Max Planck Institute for Infection Biology, Berlin, Germany; 3 University of KwaZulu-Natal, Durban, South Africa; 4 Charité Medical School, Berlin, Germany; 5 Max Planck Institute for Developmental Biology, Tübingen, Germany; University of Illinois at Chicago College of Medicine, UNITED STATES

## Abstract

Cell-to-cell spread of HIV, a directed mode of viral transmission, has been observed to be more rapid than cell-free infection. However, a mechanism for earlier onset of viral gene expression in cell-to-cell spread was previously uncharacterized. Here we used time-lapse microscopy combined with automated image analysis to quantify the timing of the onset of HIV gene expression in a fluorescent reporter cell line, as well as single cell staining for infection over time in primary cells. We compared cell-to-cell spread of HIV to cell-free infection, and limited both types of transmission to a two-hour window to minimize differences due to virus transit time to the cell. The mean time to detectable onset of viral gene expression in cell-to-cell spread was accelerated by 19% in the reporter cell line and by 35% in peripheral blood mononuclear cells relative to cell-free HIV infection. Neither factors secreted by infected cells, nor contact with infected cells in the absence of transmission, detectably changed onset. We recapitulated the earlier onset by infecting with multiple cell-free viruses per cell. Surprisingly, the acceleration in onset of viral gene expression was not explained by cooperativity between infecting virions. Instead, more rapid onset was consistent with a model where the fastest expressing virus out of the infecting virus pool sets the time for infection independently of the other co-infecting viruses.

## Author Summary

How quickly infection occurs should be an important determinant of viral fitness, but mechanisms which could accelerate the onset of viral gene expression were previously undefined. In this work we use time-lapse microscopy to quantify the timing of the HIV viral cycle and show that onset of viral gene expression can be substantially accelerated. This occurs during cell-to-cell spread of HIV, a mode of directed viral infection where multiple virions are transmitted between cells. Surprisingly, we found that neither cooperativity between infecting viruses, nor trans-acting factors from already infected cells, influence the timing of infection. Rather, we show experimentally that a more rapid onset of infection is explained by a first-past-the-post mechanism, where the fastest expressing virus out of the infecting virus pool sets the time for the onset of viral gene expression of an individual cell independently of other infections of the same cell. Fast onset of viral gene expression in cell-to-cell spread may play an important role in seeding the HIV reservoir, which rapidly makes infection irreversible.

## Introduction

Cell-to-cell spread of HIV is a mechanism of viral transmission whereby interaction between an infected donor cell and an infectable target cell leads to the directed transmission of virions to the target cell. Such interactions can occur between donor and target cells by various mechanisms [1–12], all of which involve the directed delivery of virions very close to the target cell, minimizing the distance over which virions need to diffuse and the consequent loss of virions en route [1–9, 11–24]. Because of the resulting high efficiency of viral delivery, target cells in cell-to-cell spread are exposed to multiple virions per cell both in *in vitro* infections and *in vivo* [17, 18, 25–31]. Multiple infections per cell decrease the sensitivity of cell-to-cell spread to antiretroviral drugs [17, 25, 27, 32, 33] and neutralizing antibodies [18, 34–36], and can overcome low infectivity and cellular restriction factors [37], since they increase the chances that at least one of the transmitted virions will successfully infect the cell despite inhibitors or unfavorable infection conditions [27, 38]. Because the source of insensitivity to inhibitors in cell-to-cell spread of HIV derives from multiple infections per cell, it is expected that sufficiently high inhibitor concentrations, or inhibitors more adept at suppressing multiple infections, could overcome this barrier [32, 33]. Conversely, cell-to-cell spread would offer a window of opportunity for HIV to evolve resistance to antiviral inhibitors [35].

As well as decreasing sensitivity to inhibitors, cell-to-cell spread of HIV was observed to be more rapid than cell-free infection [2, 13, 39–41]. One explanation may be fusion between donor and target cells. Fusion is insufficient for infection, as nucleic acids cannot directly infect a cell by translocating to the uninfected target cell [22]. However, the target cell would be scored as infected if a viral gene product or marker is used for detection, as fused cells share their protein pools and the marker would translocate to the target from the donor cell whether or not infection of the target cell took place. If fusion is excluded, acceleration of the viral cycle may be the result of several mechanisms: Shorter distance for the virus to transit before reaching a target cell, faster virus entry, faster pre- or post-integration dynamics due to cooperativity, and faster dynamics due to trans-acting factors secreted by the donor cells. Cooperativity would be expected to play a role in accelerating the virus cycle due to the Tat positive feedback loop [42–44], where Tat expressed from one provirus would trigger the transcript elongation of another provirus. Since the Tat protein can diffuse in and out of cells [43], such acceleration can also be potentially mediated in trans by the presence of nearby infected cells. Other HIV proteins, such as Nef, may also modify the physiology of yet uninfected cells upon cell-to-cell contact [45].

Another mechanism which can contribute to the acceleration of the viral cycle is probabilistic: since time to productive infection varies between virions due to integration site and stochastic gene expression [42, 44, 46, 47], cell-to-cell spread, which leads to multiple infections per cell, could increase the probability that at least one of the infecting viruses would have rapid infection dynamics.

Here we determined the timing of cell-to-cell spread and cell-free infection in a short infection time window, thereby limiting the role that the transit time to the target cell plays in infection timing. Despite this, we observed that cell-to-cell spread of HIV led to significantly earlier onset of viral gene expression. Surprisingly, we did not find evidence that factors secreted by donor cells, infected donor cell contact with target cells in the absence of transmission, or cooperativity between virions caused the earlier gene expression onset. We were able to replicate earlier onset in viral protein expression by increasing the multiplicity of infection with cell-free virus. This explains the observed rapid onset of viral gene expression of cell-to-cell spread by a mechanism where the fastest virus to be expressed sets the time of infection independently of other infections of the same cell.

## Results

### Cell-to-cell spread leads to earlier onset of HIV gene expression

In this study, we used the timing of the detectable onset of viral gene expression as a measure of the rate of the viral cycle. We used several ways to detect HIV gene expression, as summarized in S1 Table. Virus used for infection was produced from a molecular clone of the NL4-3 HIV strain to minimize any sequence differences between infecting virions. To compare the onset of cell-free infection to cell-to-cell spread, we infected target cells with either cell-free virus obtained from the filtered supernatant of virus producing cells, or by coculture with infected donor cells. In coculture, infection consists of a mix of cell-to-cell spread of HIV and cell-free infection. Hence, any observed difference between coculture and cell-free infection would be an underestimate of the difference between cell-to-cell spread and cell-free infection.

**Fig 1:**
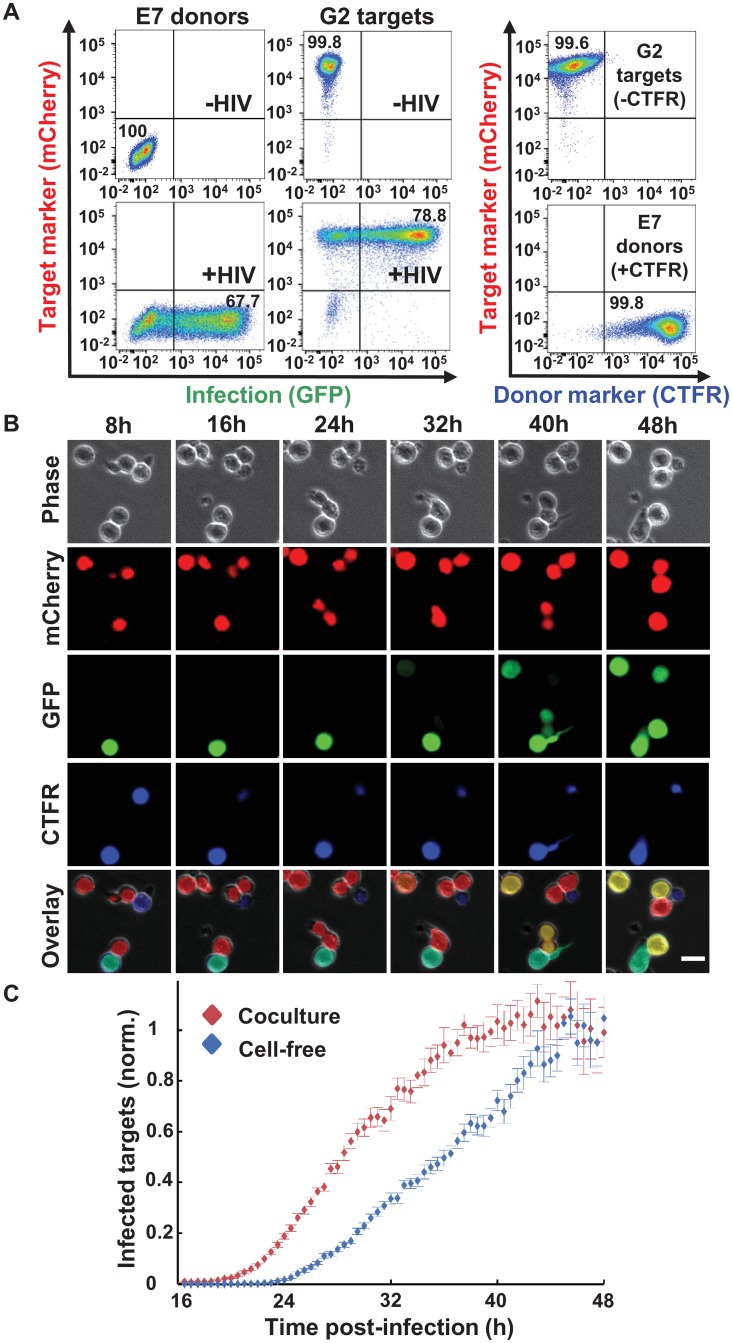
HIV cell-to-cell spread leads to earlier onset of HIV gene expression. (**A**) Infection system. Left column shows uninfected (top plot) or HIV infected (bottom plot) E7 donor cells. Middle column shows uninfected (top plot) or HIV infected (bottom plot) G2 target cells. Right column shows the labelling of donor cells with CTFR for donor-target fusion exclusion. Numbers are percent of cells in the associated quadrant. (**B**) Time-lapse images from one field of view (FOV). At each time point post-infection, cells were imaged for GFP, mCherry, and CTFR fluorescence. Bar is 15μM. (**C**) Quantified timing of coculture (red) versus cell-free (blue) infection. Each point represents the frequency of infected target cells normalized to the mean frequency in the last three hours of the movie. Shown are means and standard errors over 25 FOVs. One of five independent experiments.

In order to quantify the onset of coculture versus cell-free infection by time-lapse microscopy, we imaged infection in the RevCEM cell line [48]. This cell line contains a GFP reporter that is responsive to the HIV splicing regulator protein Rev and hence reflects the timing of late HIV proteins [43, 49–51]. In order to efficiently detect infection, we subcloned the cell line to produce the reporter clone E7. This increased the maximum percentage of GFP positive cells from approximately 10% in the parental line to 70% in E7 (Fig 1A, left column). To enable the automated determination of the number of infected target cells (S1 Fig), we further stably expressed mCherry in these cells and derived the mCherry labelled G2 clone (Fig 1A, middle column). To exclude donor-target cell fusions, we labelled donor cells with the vital stain CellTrace Far Red (CTFR, Fig 1A, right column). CTFR and mCherry double positive cells were excluded from the analysis. In the absence of fusion exclusion, coculture infection showed a baseline from the earliest time points, which may not be real infection (S2 Fig).

We imaged infection over two days (S1 Movie). We used automated image analysis to determine the number of GFP^+^/mCherry^+^/CTFR^-^ cells over the total number of mCherry^+^/CTFR^-^ cells in each field of view at each frame of the movie (Fig 1B). In this experiment and the other time-lapse experiments performed in this study, we did not track individual cells, but rather measured the number of target cells with detectable viral gene expression at each time-point. We limited infection to the first two hours by washing away cell-free virus after that time window, and inhibiting additional infection cycles by addition of the protease inhibitor atazanavir (ATV), which has been described to effectively inhibit cell-to-cell transmission [33]. We imaged infection after washing and ATV addition. The protease inhibitor was used at a concentration that blocked over 99% of coculture infections (S3A Fig). This window for infection limited the time that the virus could transit to the target cell to no more than two hours in both coculture and cell-free infection. We calibrated the input of cell-free virus and infected cells so that the frequency of infected target cells after 48 hours was similar between the infection modes and did not saturate the available target cells (S4 Fig).

We quantified the fraction of infected cells over time and observed that both cell-free and coculture infection resulted in a variable time to Rev activity in individual infected cells, consistent with previous results showing heterogeneity in the length of the HIV replication cycle in cell-free infection [52]. In both infection modes, no Rev activity was detected before approximately 20 hours, corresponding to a period of intracellular delay [53–55]. On average, coculture infection showed more rapid HIV gene expression relative to cell-free infection (Fig 1C). We derived the mean and standard deviation for the timing of coculture and cell-free infections by parametrizing the number of infected cells over time with a best fit Gamma distribution, since Gamma distributions are a standard model for the timing of multi-step processes [56]. We obtained a time to detectable per cell Rev activity in coculture infection of 28±5.0 hours (mean±std). In contrast, mean time to per cell Rev activity in cell-free infection was 34.5±6.1 hours. This constituted an acceleration of 19% in the mean time to Rev activity in coculture infection. The difference between the two means was significant (p = 9x10^-4^, bootstrap).

### Secreted factors and contact with infected cells in the absence of transmission do not accelerate HIV gene expression

**Fig 2:**
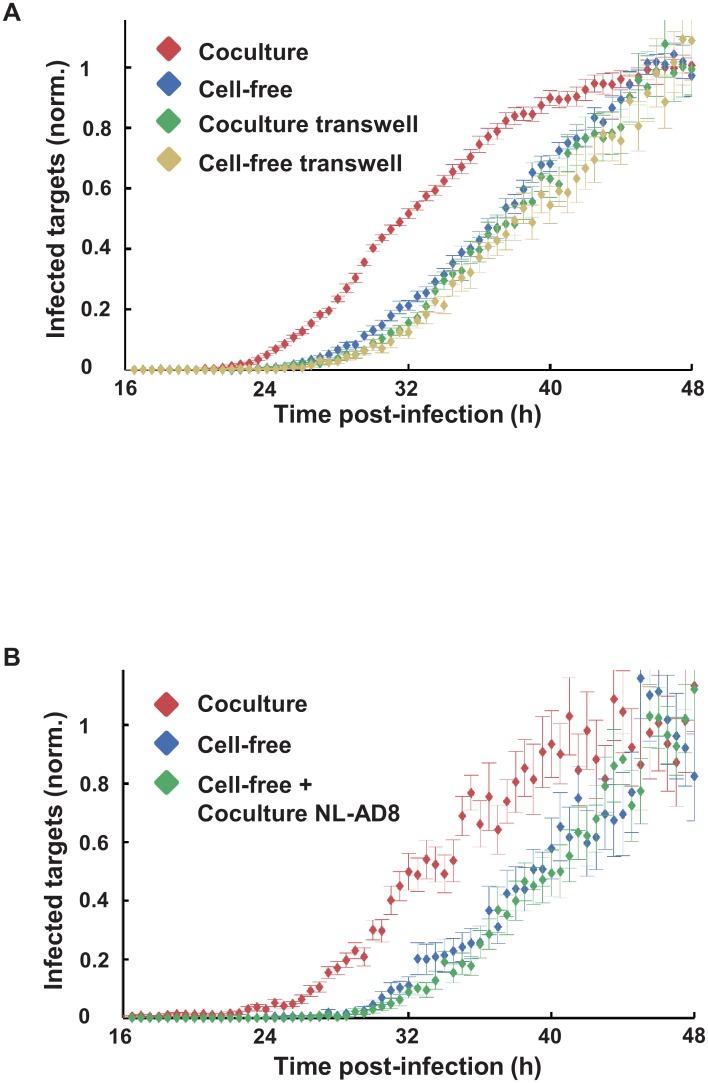
Acceleration of viral gene expression in coculture infection is not due to factors acting in trans. (**A**) Frequency of infected G2 target cells in coculture (red), cell-free (blue), coculture separated by transwell (green) or cell-free infection across the transwell (yellow). Shown are means and standard errors over 15 FOVs. One of three independent experiments. (**B**) Frequency of infected G2 target cells in coculture (red) versus cell-free infection in the absence (blue) or presence (green) of CD4^+^ primary T cells infected with NL-AD8 CCR5 tropic HIV which cannot infect G2 cells. Shown are means and standard errors over 25 FOVs. One of three independent experiments. For both experiments, frequencies of infected cells were normalized by mean infected target cell frequency in the last 3 hours of the movie. Fusion events leading to false positive target cells were excluded with CTFR.

We investigated the role of secreted factors acting in trans in the earlier onset of HIV gene expression by coculture with infected donor cells. To isolate the contribution of factors acting in trans, we separated infected donors from targets by a transwell membrane permeable to cell-free virus and soluble factors. We obtained no acceleration of time to detectable GFP expression using transwell infection (Fig 2A).

We considered the possibility that factors acting in trans may only operate over very short distances or that direct contact between donor and target cells, unrelated to viral transmission, may be required for an earlier onset of HIV gene expression. To test this, we took advantage of the fact that our reporter cell line could only be infected with HIV which uses the CXCR4 co-receptor. We therefore infected cells using the cell-free route with our CXCR4 tropic strain (NL4-3) in the presence of cocultured CD4^+^ cells infected with CCR5 tropic HIV (NL-AD8). This CCR5 tropic strain is identical to NL4-3, except for the Env protein, which is specific for the CCR5 co-receptor. We verified that NL-AD8 infected CD4^+^ cells could not infect the G2 target cells by coculture (S5 Fig). We did not observe a more rapid onset of HIV gene expression of cell-free infection cocultured with cells infected with the CCR5 tropic HIV compared to cell-free infection in the absence of these cells (Fig 2B), indicating that trans-acting factors are unlikely to induce an earlier onset of viral gene expression.

### Multiple infections per cell result in earlier onset of HIV gene expression

We asked whether the higher force of infection in cell-to-cell spread, manifesting as multiple infections per target cell, leads to earlier onset of HIV gene expression. We therefore used concentrated cell-free virus to mimic the higher infection levels per cell observed in cell-to-cell spread. We used the highly infection permissive MT4 cell line [27] to enable infection at a multiplicity greater than 1 within a two-hour infection window. As a reporter for infection, we used the NL4-3YFP strain of HIV [57] which substitutes YFP for the HIV early gene Nef. Therefore, YFP expression reflects the timing of HIV early genes (S1 Table). We infected MT4 cells with NL4-3YFP cell-free virus (S2 Movie) at increasing multiplicities of infection (MOI) per target cell, starting at an MOI of 0.1 infectious units per cell and up to an MOI of 4. After two hours, we removed the residual virus by washing and added sufficient ATV to prevent additional infections from coculture (S3B Fig).

**Fig 3:**
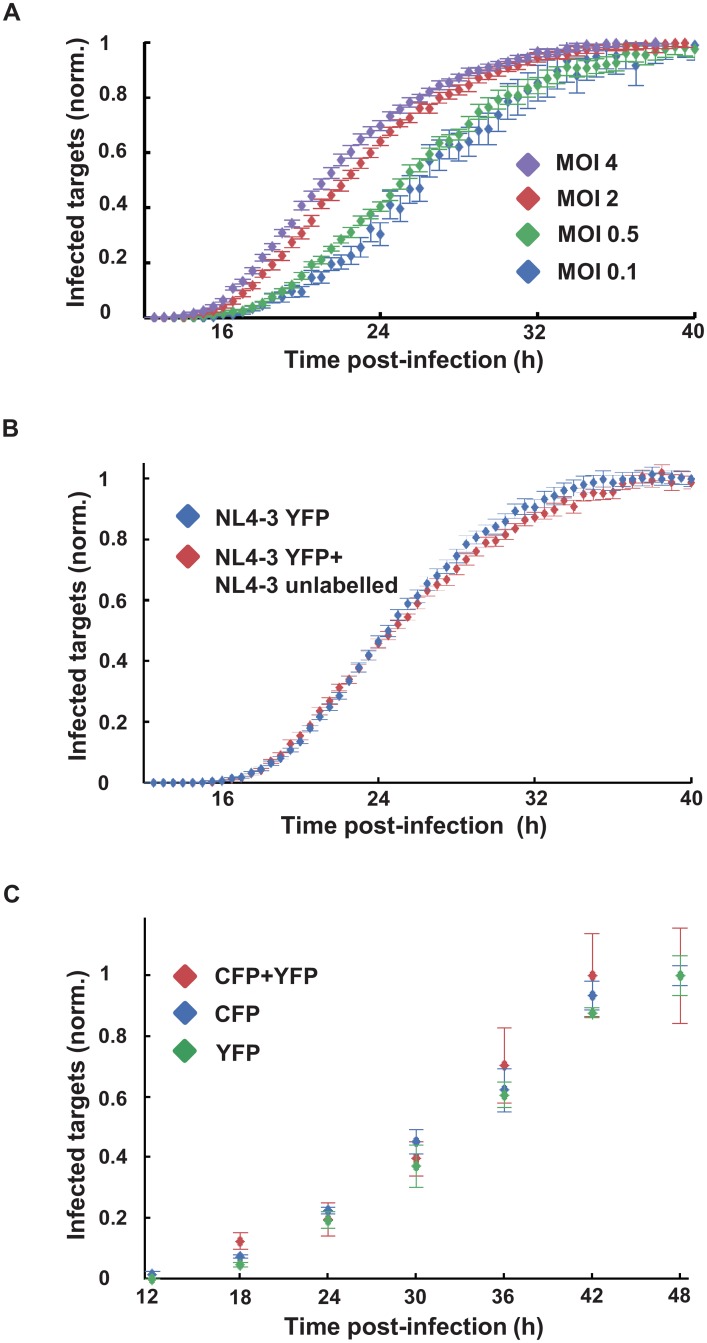
Earlier onset of HIV gene expression by multiple infections per cell. (**A**) Timing of HIV gene expression onset as a function of multiplicity of infection (MOI) per cell. MT4 cells were infected with HIV strain NL4-3YFP at an MOI of 0.1 (blue), 0.5 (green), 2 (red) and 4 (purple). Shown are means and standard errors over 15 FOVs. One of three independent experiments. (**B**) Measurement of cooperativity between infecting viruses. Infection as detected using YFP expression when cells were infected with NL4-3YFP alone at MOI of 0.3 (blue), or with YFP at the same MOI co-infected with unlabeled NL4-3 at an MOI of 8 (red). Shown are means and standard errors over 25 FOVs. One of three independent experiments. For both experiments, frequencies of infected cells were normalized by mean infected cell frequency in the last 3 hours of the movie. (**C**) Measurement of cooperativity in onset of HIV gene expression in CD4^+^ T cell infection. Cells were infected with NL4-3YFP and NL4-3CFP, and single (CFP or YFP) and double infected (CFP+YFP) cells were quantified by flow cytometry at the indicated time points. The frequency of CFP, YFP, and CFP+YFP positive cells normalized to the frequency at 48 hours post-infection is indicated by blue, green, and red points respectively. Shown are mean and standard errors of quadruplicate measurements of one representative experiment out of three, using three different blood donors.

We observed a more rapid onset of YFP expression with increasing MOI, accelerating mean expression time from 27.5±6.1 hours at an MOI of 0.1, which results almost exclusively in infections with one virus, to 22.6±5.5 hours at an MOI of 4 (Fig 3A). This acceleration in onset relative to the 0.1 MOI infection was significant (p = 1.7x10^-3^ for MOI = 0.5, p<10^−4^ for MOI = 2 and MOI = 4 using bootstrap).

We asked whether this earlier onset was mediated by cooperativity: pre- or post-integration interactions between virions that would lead to faster HIV gene expression. For this, we compared MT4 cells infected with NL4-3YFP alone to MT4 cells co-infected with NL4-3YFP and the unlabeled NL4-3 strain of HIV. The unlabeled HIV infection was at high multiplicity (MOI = 8) to ensure that the majority of cells infected with the YFP reporter HIV were also co-infected with the unlabeled HIV. If cooperativity has a role in the more rapid onset of viral gene expression, the unlabeled virus should accelerate the expression of labelled virus to the threshold of detection. However, we observed that co-infection did not lead to a more rapid onset of YFP expression (Fig 3B).

MT4 cells are known to be infected with HTLV-I [58] and hence any lack of cooperativity due to co-infection may be the result of saturating cooperativity with the endogenous virus. We therefore proceeded to investigate cooperativity in the onset of HIV gene expression between co-infecting viruses in primary CD4^+^ T cells. To investigate cooperativity in this system, we co-infected cells by the cell-free route with HIV expressing YFP and HIV expressing CFP. We detected the number of infected cells by flow cytometry at 6 hour intervals. We obtained CFP and YFP singly infected cells, as well as low but significant numbers of double infected cells (S6 Fig). We did not observe differences in timing of the onset of viral gene expression between singly infected and the CFP/YFP co-infected cells, indicating that co-infecting viruses did not show cooperativity in the onset of viral gene expression in primary CD4^+^ T cells and confirming our results in MT4 cells.

**Fig 4:**
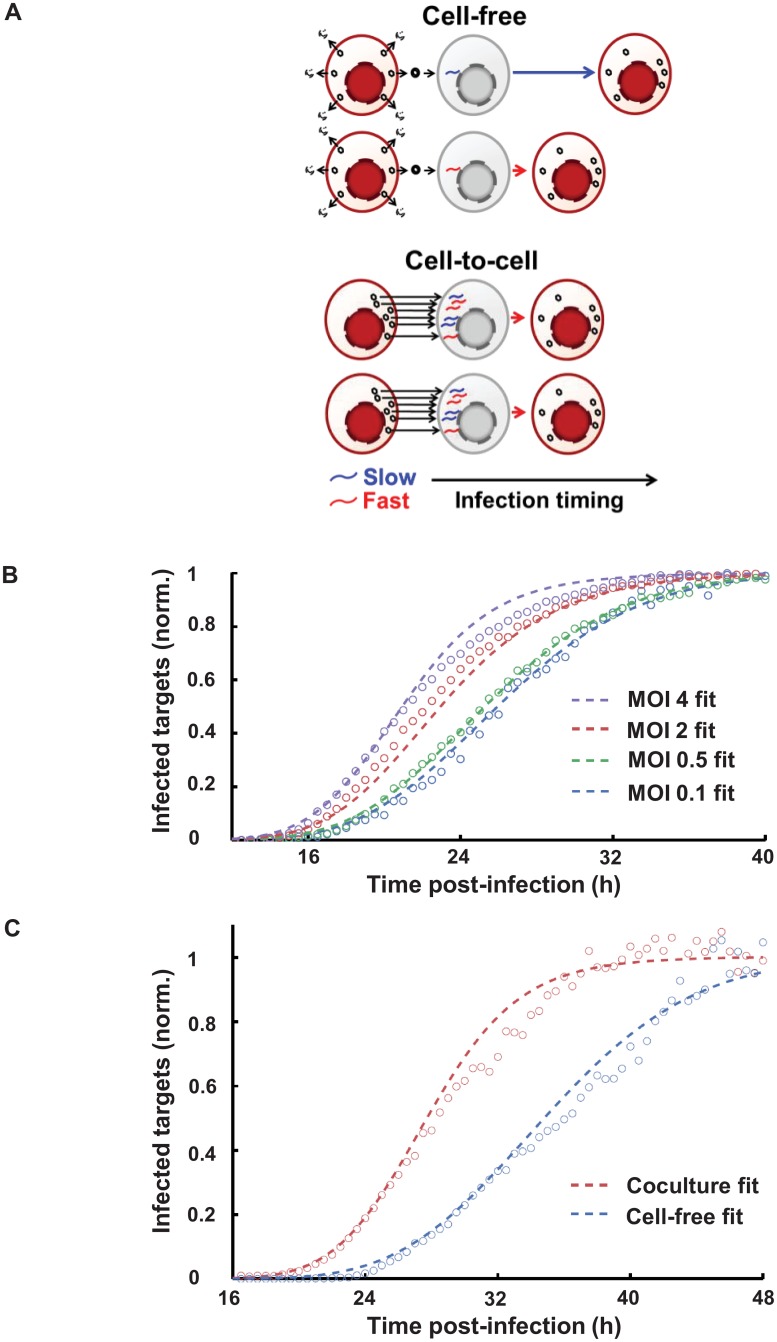
Modelling faster onset of HIV gene expression by multiple infections per cell. (**A**) Proposed probabilistic mechanism for the more rapid onset of HIV gene expression. (**B**) Simulation of cell-free infection times at different MOI by random draws from a Gamma distribution parametrizing the best fit cell-free infection times of single virion infections. Circles are experimental data from Fig 3A, dashed lines represent the simulation results for MOI of 0.1 (blue), 0.5 (green), 2 (red), and 4 (purple). Fitted means±std of infection times were 26.4±5.2, 25.2±5.1, 22.7±4.8, and 21.9±4.9 hours. (**C**) Fit of coculture versus cell-free infection. Circles are experimental data from Fig 1C, dashed lines represent the simulation results. Means±std for the fits were 28.0±5.0 hours for coculture and 34.5±6.2 hours for cell-free infections. Best fit MOI for the coculture infection was 4.6.

Since cooperativity between virions could not account for the earlier onset of HIV gene expression, we asked whether multiple infections per cell accelerated onset of gene expression by a first-past-the-post mechanism, where the earliest virus to express sets the time of infection (Fig 4A). This mechanism operates if: 1) Each integrated virus has a stochastically set time to viral protein expression. 2) Infections proceed independently. 3) A single expressed virion is sufficient to make use of target cell resources so that the target cell becomes infectious [26].

We reasoned that if a cell is infected by *n>1* virions, the virion that first completes the replication cycle sets the time to infection. If each infection is independent, the distribution of the time to infection given *n* virions per cell is:

$$p\left(t,n\right)=n\;p\left(t\right){\left(1-Q\left(t\right)\right)}^{n-1}.$$ (1)

Here *p(t)* is the distribution of the time to viral gene expression given a single virion per cell approximated by a Gamma distribution, and *Q(t)* is the corresponding cumulative distribution. In an infection with an average MOI *m*, the cells will be infected with a number of virions which is Poisson distributed around *m* and can be modelled by the average of Eq 1 over different *n* with Poisson weights (excluding *n = 0*). The distribution of the time to viral gene expression at *m* is then given by:

$$\rho \left(t,m\right)=\frac{e^{-m}}{1-e^{-m}}\Sigma_{n=1}\frac{m^{n}}{n!}p\left(t,n\right),$$ (2)

where the pre-factor normalizes the distribution. We determined the shape and scale parameters of *p(t)* by jointly fitting the time series data for the multiple MOI infections to Eq 2. The model fits the data well for MOI 0.1, 0.5, 2, 4 with only the two parameters of the Gamma distribution, indicating that our model of independent stochastic infections can explain the acceleration at high MOI (Fig 4B).

To determine the effective MOI for coculture infections, we fitted the time course data to Eq 2 using the shape and scale parameters of the Gamma distribution determined by a fit to the cell-free data, approximating *n = 1* for cell-free infections (Fig 4C). We obtained that the acceleration of viral gene expression with coculture was predicted by an effective MOI of 4.6 per cell.

### Coculture infection leads to earlier onset of HIV gene expression in primary human cells

**Fig 5:**
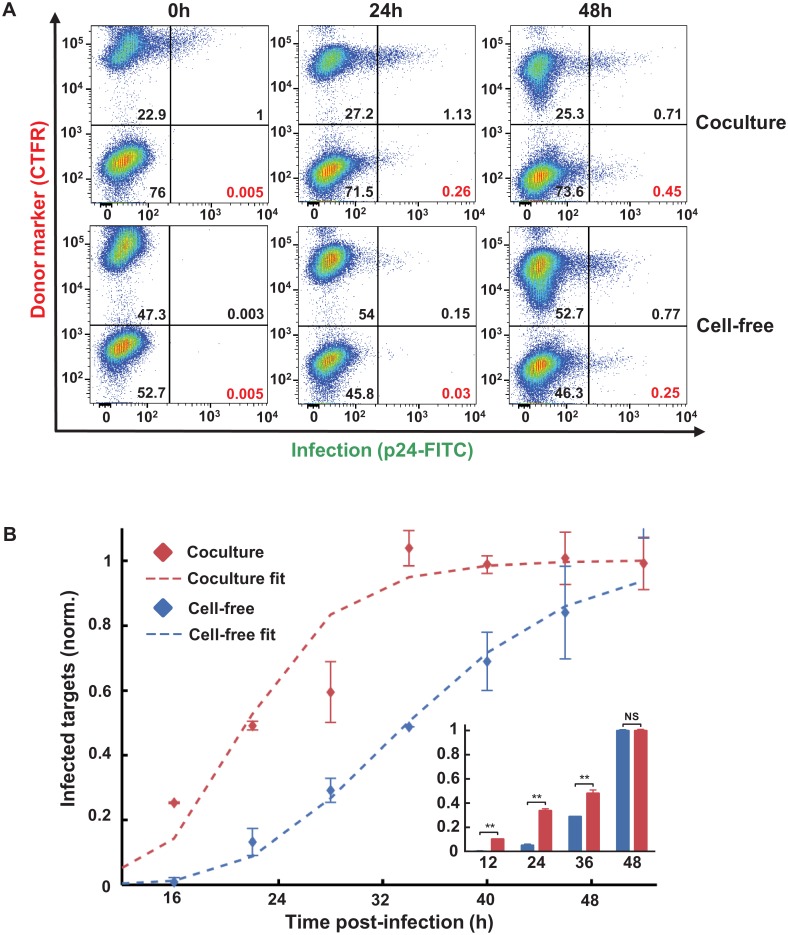
Earlier onset of HIV gene expression in PBMCs by coculture infection. (**A**) Gating strategy for detectably infected target cell frequency. Donors were labelled with CTFR and infection was assayed by flow cytometry following p24 staining for HIV Gag. Top row is coculture infection, bottom row is cell-free infection. Percent of infected targets in the population (bottom right quadrant) shown in red, and values for other subpopulations in black. (**B**) Timing of coculture (red) versus cell-free (blue) infection. Shown are means and standard errors of duplicates from one of three experiments with different blood donors. Frequencies of infected target cells were normalized by infected target cell frequency at 50 hours post infection. Means±std of best fit Gamma distributions were 22.1±9.3 hours for coculture and 34.2±9.1 hours for cell-free infection. Best fit MOI for the coculture infection was 5.0 (dashed red line). Inset: Infection of purified CD4^+^ T cells by cell-free HIV or autologous infected CD4^+^ T cells. Shown are mean and standard errors of duplicates of the frequencies of infected target cells normalized to the frequency of infected target cells at 48 hours post-infection. One of three experiments, each performed on purified CD4^+^ T cells from a different individual. Timing differences between cell-free and coculture in CD4^+^ T cells were statistically significant at 12, 24 and 36 hours post-infection (p<0.01, two-tailed unpaired t-test corrected for multiple comparisons using the Sidak-Bonferroni method).

To examine whether the earlier onset of HIV gene expression observed in the cell line also occurs in primary cells, we used coculture with autologous infected donor cells or cell-free virus to infect peripheral blood mononuclear cells (PBMCs) derived from healthy donors. As with the cell lines, donor cells were separated from target cells by labelling them with a vital stain. The fraction of infected target cells at different times post-infection was quantified by detection of the viral p24 protein, made as part of the HIV Gag polyprotein, using flow cytometry (Fig 5A). Virus was washed away after 2 hours in both cell-free and coculture infections, and ATV added to prevent additional infection cycles. ATV was used at a concentration sufficient to inhibit more than 99% of coculture infections (S3C Fig). Coculture dramatically accelerated the onset of HIV gene expression as measured by the detection of the HIV Gag protein relative to cell-free infection in primary human cells from 34.2±9.1 hours to 22.1±9.3 hours (mean±std). This constituted a decrease of 35% in the mean time to detectable HIV Gag expression (Fig 5B). Based on the cell-free distribution, the best-fit MOI per cell in coculture infection to recapitulate the difference in viral expression onset was 5.0 (Fig 5B, dashed red line).

PBMCs contain monocytes and other cells which may complicate interpretation of these results. To investigate whether T cell to T cell transmission was sufficient for the faster onset of viral gene expression, we repeated the experiment with purified CD4^+^ T cells (Fig 5B Inset and S7 Fig). We confirmed that cell-to-cell transmission between autologous T cells resulted in a more rapid onset of viral gene expression relative to cell-free infection.

**Fig 6:**
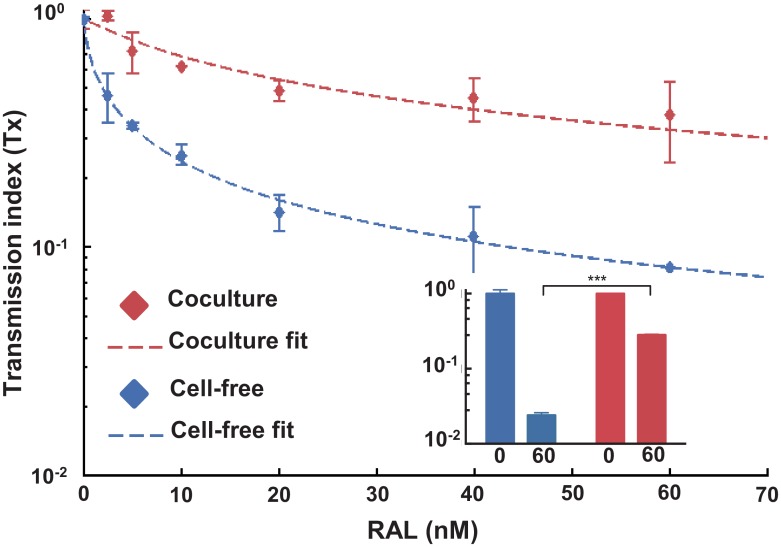
Decreased sensitivity of coculture HIV spread to RAL predicts multiple infections per cell. Sensitivity of coculture (red) and cell-free (blue) infection to the integrase inhibitor raltegravir (RAL) in PBMCs. Points are means and standard errors of duplicates from one of three experiments with different blood donors. Transmission index (Tx) was calculated as the number of infected target cells with RAL divided by the number of infected target cells without RAL. Blue dashed line represents parametrization of the cell-free infection response to RAL in terms of IC_50_ (1.9 nM) and hill coefficient (0.7). Red dashed line represents the fit of coculture infection using the decreased sensitivity to RAL (Materials and methods). An effective MOI of 4.8 is predicted for coculture infection. Inset: Sensitivity of primary CD4^+^ T cell infection to RAL. Points are Tx means and standard errors of duplicates from one of three experiments with different blood donors. The drug sensitivity differences in CD4^+^ T cells between cell-free and coculture was statistically significant (p < 0.001, two-tailed unpaired t-test).

We next proceeded to compare our predicted number of infections per cell using the timing of the onset of viral gene expression to that obtained by a second method. We have previously developed an approach to predict the number of infections per cell in cell-to-cell spread based on the reduced sensitivity to antiretroviral drugs relative to cell-free infection [27]. We therefore performed the PBMC infection in the presence of the integrase inhibitor raltegravir (RAL). As in the timing experiments, we used a 2-hour infection window. Coculture infection decreased sensitivity to RAL (Fig 6), consistent with our previous work and that of others showing that cell-to-cell spread decreases sensitivity to HIV inhibitors. For PBMC infection, IC_50_ of cell-free infection was 1.9nM and the maximum concentration of RAL used (60nM) decreased infection 12.2-fold. In contrast, IC_50_ of infection was 10nM and infection was reduced 2.6-fold at the same RAL concentration when transmission was by coculture. Reduced RAL sensitivity of coculture infection was also confirmed with transmission between purified autologous CD4^+^ T cells using a 2-hour infection window (Fig 6 Inset). In this case, 60nM RAL reduced cell-free infection by 33.3-fold. In contrast, coculture infection was reduced 3.6-fold. The best-fit MOI per cell needed to account for the reduced sensitivity of PBMC coculture infection to RAL was 4.8, which was similar to the number of virions predicted using infection timing under the same infection conditions.

## Discussion

We have observed faster onset of viral gene expression in coculture infection containing cell-to-cell spread of HIV relative to cell-free HIV infection. The earlier onset of viral gene expression in coculture was lost when target cells were separated from donor cells by a transwell membrane. A faster virus cycle in cell-to-cell spread relative to the non-directed, cell-free mode of infection has been previously observed directly [2, 13, 41] and inferred through modelling of infection dynamics [39, 40]. Here we used time-lapse microscopy of HIV infection to directly quantify and investigate the mechanism behind the faster onset of viral gene expression. We minimized possible differences between cell-to-cell spread and cell-free infection in the extracellular transit time from donor to target cell by limiting the time window of transmission to 2 hours. We have also minimized any contribution of virus sequence to different viral gene expression dynamics by using viruses with identical sequences derived from a molecular clone. Hence, variability in gene expression is a result of the interaction of the virus with the host cell. After exclusion of donor-target cell fusions, we found a minimum time for early viral protein expression in both infection modes, corresponding to a period of intracellular delay indicative of true infection [53–55]. We found that we could recapitulate the faster onset of viral gene expression by increasing the MOI of cell-free virus, and that there was no evidence for cooperativity or interference between co-infecting viruses. There was also no evidence for trans-acceleration of HIV gene expression onset from the surrounding infected cells.

Previous studies on cell-to-cell spread have concentrated on understanding the mechanisms by which cell-to-cell transmission occurs, and such mechanisms may lead to a faster onset of the expression of viral genes in the infected target cell in addition to making the infection more efficient. For example, it has been reported that the infected donor cell rapidly polarizes to the site of contact with the target cell [20] and that the subsequent transmission to the target cell occurs quickly [4, 18, 59, 60], though viral membrane fusion has been reported to be slower in cell-to-cell spread relative to cell-free infection [16]. Hence, a faster onset of HIV gene expression in cell-to-cell spread may be strictly mechanistic, due to more rapid entry of the virus. In this case, it would be expected that increasing cell-free MOI would not lead to faster onset, as increasing the MOI does not change the attachment and entry route. Since our data shows that cell-free MOI does control the onset of HIV gene expression, mechanistic factors such as more rapid entry in cell-to-cell spread are unlikely to play a major role.

Given multiple infections of the same cell in cell-to-cell spread, we would expect three possibilities of how co-infecting viruses could interact at the level of viral gene expression [61]. The first would be synergistic/cooperative interactions, where expression of one virus amplifies the expression of a co-infecting virus. The second would be no interaction, and the third would be that co-infecting viruses may compete for cellular resources and hence expression of one virus would interfere with the expression of co-infecting viruses. For example, comparing 10 co-infecting viruses to 10 infections identical in every way except occurring in 10 different cells, cooperativity would lead to the cell with 10 co-infecting viruses to show more rapid onset of viral gene expression relative to any one of the 10 single infections. No interaction between viruses would lead to the onset in the cell with 10 co-infecting viruses to be as fast as the fastest cell among the 10 singly infected cells, what we term a first-past-the-post mechanism. Interference or antagonism would lead to the cell with 10 co-infecting viruses to show a slower onset of viral gene expression than the fastest cell among the 10 singly infected cells, and possibly slower than the other singly infected cells.

Interactions between co-infecting viral genomes in HIV and other viruses have been extensively documented. For example, co-infecting viruses share post-integration components by a process known as complementation [62–66]. Hence, we would have predicted that there is at least some cooperativity in viral gene expression between co-infecting viruses as a result of the Tat positive feedback loop, as intracellular Tat concentration should increase with the number of expressed proviruses [42–44]. This mechanism of cooperativity would be expected to manifest as faster onset of viral gene expression since the delay to build up Tat levels by basal transcription should be reduced [44]. However, no detectable differences in the timing of the onset of HIV gene expression upon co-infection of YFP-HIV with unlabeled virus and no detectable differences when primary CD4^+^ cells were co-infected with two viruses argues against the presence of cooperativity at the onset of gene expression. Likewise, no interference was observed. Instead, we found that the mechanism most consistent with the faster onset of viral gene expression was that multiple infections per cell in coculture infection resulted in a pool of viruses which express viral genes at different times post-infection. The virus with the fastest onset of gene expression from this pool sets the start time for the generation of viral components by the infected cell.

We note that the lack of cooperativity as detected at the onset of HIV gene expression does not mean that co-infecting viruses do not interact, and interactions may occur later in the virus cycle. For example, interference may be expected to occur close to the time of peak virus production, where co-infecting viruses could compete for limited cellular resources to assemble virions [26, 67]. Such effects would influence the number of virions produced, but not the onset of gene expression as measured here.

We compared the predicted number of infections per cell based on the timing of the viral cycle to that predicted by the decreased sensitivity of coculture infection to an antiretroviral drug. The MOI per cell in PBMC coculture infection was predicted by timing to be 5.0 infectious viruses. This was similar to the predicted MOI based on the degree of insensitivity of PBMC coculture infection to the antiretroviral RAL (MOI = 4.8). Interestingly, the drug insensitivity of cell-to-cell spread to RAL was maintained despite keeping infection to one virus cycle using ATV. This indicates that the faster virus cycle of cell-to-cell spread is not necessary for drug insensitivity. However, a faster virus cycle may contribute to replication in the face of drug by amplifying an expanding infection.

Assuming that faster viral gene expression leads to more rapid viral dynamics, a more rapid onset of the viral cycle may confer a fitness advantage of rapid initial expansion, or transmission where the turnover rate of infected cells is high [2, 68]. Reasons for high turnover may include targeting of infected cells by cytotoxic T lymphocytes [69, 70], or a limited infection window due to bystander killing of target cells [71–74], all operating in environments such as lymph nodes where cell-to-cell infection is likely to occur [29, 31, 71, 73]. In exponential expansion at the R_0_ observed during primary HIV infection (~8, [75]), decreasing the infection cycle time by one quarter can lead to a 2 order of magnitude increase in the number of infected cells over several weeks. A large reservoir would be a barrier to a prolonged period of treatment interruption without rebound or to a permanent cure [76–81]. Thus, a faster viral cycle may seed a larger HIV reservoir, which would be more difficult to eliminate. However, if the most rapid virus cycle rate gives the highest fitness advantage, then cooperativity in gene expression would have been expected to evolve. Yet it does not seem to occur, perhaps indicating drawbacks to cooperativity such as more rapid cytotoxicity, or decreased ability of the virus to enter a quiescent state [82–85].

## Materials and Methods

### Ethical statement

Blood was obtained from adult healthy volunteers after written informed consent (University of KwaZulu-Natal Institutional Review Board approval BE022/13).

### Inhibitors, viruses and cells

The following reagents were obtained through the AIDS Research and Reference Reagent Program, National Institute of Allergy and Infectious Diseases, National Institutes of Health: the antiretroviral drugs ATV and RAL; Rev-CEM cells from Y. Wu and J. Marsh [48]; MT-4 cells from D. Richman [58]; HIV expression plasmid pNL4-3 from M. Martin [86] and pNL-AD8 from E. Freed [87]. The NL4-3YFP molecular clone was a gift from D. Levy [57]. Cell-free viruses were produced by transfection of HEK293 cells (ATCC) with molecular clones using TransIT-LT1 (Mirus) or Fugene HD (Roche) transfection reagents. Supernatant containing released virus was harvested after two days of incubation and filtered through a 0.45μm filter (Corning). The number of virus genomes in viral stocks was determined using the RealTime HIV-1 viral load test (Abbott Diagnostics). To produce the E7 clone, RevCEM cells were subcloned at single cell density and screened for the fraction of GFP expressing cells upon HIV infection using microscopy. To produce the G2 clone, E7 cells were stably infected with the mCherry gene under the control of the EF-1α promoter on a pHAGE2 based lentiviral vector (gift from A. Balazs), subcloned, and screened for clones with >99% mCherry positive cells. Similarly, the MT4-mCherry cell line was created by infecting MT4-cells with the pHAGE2 lentiviral vector expressing mCherry. PBMCs were isolated by density gradient centrifugation using Histopaque 1077 (Sigma-Aldrich). CD4^+^ cells were positively selected using CD4 Microbeads loaded onto MACS separation columns according to manufacturer’s instructions (Miltenyi Biotec). Culture and experiments were performed in complete RPMI 1640 medium supplemented with L-Glutamine, sodium pyruvate, HEPES, non-essential amino acids (Lonza), and 10% heat-inactivated FBS (Hyclone). Primary cells were additionally supplemented with IL-2 at 5ng/ml (PeproTech). PBMCs and CD4^+^ T cells were activated at 2*10^6^ per ml density for one (donor cells) or three days (target cells) with PHA at 10μg/ml (Sigma-Aldrich).

### Infection

For infection of RevCEM clones, 5x10^5^ cells/ml E7 reporters were infected with 2x10^8^ NL4-3 viral copies/ml (20ng p24 equivalent [88]) and used as infected donor cells. Infected and uninfected donors were incubated for two days, then stained with CellTrace Far Red (CTFR, Thermo Fisher Scientific) at 1μM and washed according to manufacturer’s instructions. G2 reporters at 5x10^5^ cells/ml were either cocultured with 1:20 infected donor cells, or 1:20 uninfected donor cells and 10^9^ NL4-3 viral copies/ml cell free virus. For RevCEM coculture experiments with cells infected with CCR5 tropic HIV, activated CD4^+^ cells at a concentration of 10^6^ cells/ml were infected with 2x10^8^ NL-AD8 viral copies per ml. Infected and uninfected CD4^+^ cells were incubated for two days. After two days, CD4^+^ cells were stained with CTFR as above. G2 cells were then infected with 10^9^ copies/ml cell-free NL4-3, and cocultured with either infected or uninfected CD4^+^ cells, equal in number to the number of NL4-3 infected E7 cells added to the coculture positive control. For MT4 infections, cells were infected at a density of 5x10^5^ cells/ml with 1.2x10^8^ (MOI = 0.1) to 5x10^9^ (MOI = 4) viral copies per ml of NL4-3YFP. For cooperativity experiments, MT4 cells were infected with 4x10^8^ NL4-3YFP alone (MOI = 0.3) or co-infected with 4x10^8^ copies of NL4-3YFP (MOI = 0.3) and 5x10^9^ copies NL4-3 (MOI = 8). For PBMC infections, one day activated cells at a concentration of 10^6^ cells/ml were used as donors and infected with 2x10^8^ NL4-3 viral copies per ml. Donor cells were incubated for two days, and were separated from target cells by labelling them with CTFR or with carboxyfluorescein succinimidyl ester at 1μM (CFSE, Thermo Fisher Scientific) vital stain. CTFR or CFSE positive cells were excluded from the analysis, being either donors or donor-target fusions. Three day activated PBMC target cells at 10^6^ cells/ml were then infected with either 1:10 infected donor cells, or with 1:10 uninfected donor cells and 5x10^8^ copies of cell-free NL4-3. All cell-free and coculture infections of target cells were washed twice in medium after a two hour incubation with cell-free virus or infected donors, then resuspended in fresh growth medium with ATV. In the RAL sensitivity experiments, RAL was pre-incubated with target cells 4 hours before infection. Experiments comparing drug sensitivity and viral expression onset of co-culture and cell-free infections in primary CD4^+^ T cells were performed as with PBMCs. For experiments examining cooperativity in CD4^+^ T cells, infection with NL4-3YFP and NL4-3CFP was performed by adding 5x10^8^ cell-free virions of each strain per 10^6^ cells. CD4^+^ T cells were washed twice 2 hours post-infection and ATV was added as for PBMCs.

### Staining and flow cytometry

PBMCs and CD4+ cells infected with NL4-3wt or NL-AD8 were strained with anti-p24 FITC-conjugated or PE-conjugated antibody (KC57, Beckman Coulter) using the Cytofix/Cytoperm and the Perm/Wash buffers (BD Biosciences) according to manufacturer’s instructions. Cells were acquired with a FACSAriaIII or FACSCaliber machine (BD Biosciences) using 488 and 640nm laser lines. A minimum of 10^5^ cells per sample were acquired. Results were analyzed with FlowJo 10.0.8 software. For CFP/YFP co-infection experiments, cells were acquired with a FACSAriaIII using the 405nm laser line for CFP, and 488nm laser line for YFP.

### Time-lapse microscopy

Cell density was reduced to 7x10^4^ cells/ml and cells were attached to ploy-l-lysine (Sigma-Aldrich) coated 6-well optical plates (MatTek). Cell-free and coculture infections were imaged in tandem using a Metamorph-controlled Nikon TiE motorized microscope with a 20x, 0.75 NA phase objective in a biosafety level 3 facility. Excitation sources were 488 (GFP, YFP), 561 (mCherry), or 640 nm (CTFR) laser lines and emission was detected through a Semrock Brightline quad band 440–40 /521-21/607-34/700-45 nm filter. Images were captured using an 888 EMCCD camera (Andor). Temperature (37°C), humidity and CO_2_ (5%) were controlled using an environmental chamber (OKO Labs). Fields of view were captured every 30 minutes and a minimum of 1000 target cells were acquired per condition. Threshold for detection of the onset of HIV gene expression was set so that no positive cells were detected in the uninfected control. Cells with above threshold expression were scored as positive.

### Transwell assay

Cells were either infected by coculture in the lower compartment of a 6-well transwell plate with 0.4 μm pores (Costar) or separated across the membrane. To maintain a similar fraction of infected cells, 10-fold more donors were used when infection was across the membrane relative to coculture. Cell-free infection was performed in the lower compartment or across the membrane. After six-hour incubation, infection was washed, ATV added, and cells transferred to optical plates for imaging, keeping the donors in their initial compartments but not in the focal plane.

### Image analysis

Movies were analyzed using custom code developed with the Matlab R2014a Image Analysis Toolbox. Images in the mCherry channel were thresholded to obtain images, and the imfindcircle function used to detect round objects within the cell radius range. Cell centers were found. GFP and CTFR signals underwent the same binary thresholding. The number of mCherry positive 16 pixel^2^ squares around the cell centers, negative for fluorescence in the CTFR channel and positive for fluorescence in the GFP channel, was used as the number of infected target cells. YFP signal in MT4 mCherry cells was analyzed in the same way except no CTFR stain was used, as infection was by cell-free virus.

### Normalization to distribution tail

For time-lapse experiments, data was normalized to compare infection between experimental conditions that had a similar, but not exactly equal number of infected cells. Normalization was by the average of the fraction of infected target cells during the last three hours to accurately capture the maximum infection level at the end of the viral cycle. Normalization by the maximum number of infected target cells was found to be noisy since it was sensitive to outlier values in the data.

### Modeling

Fitting of time-lapse data was done using a custom Python script using the Powell minimization algorithm from scipy (S1 Script). For drug sensitivity modelling, cell-free infection in the presence of increasing RAL concentrations was parametrized using the relation

$$d=1-\frac{1}{1+{\left(\frac{IC_{50}}{D}\right)}^{h}},$$ (3)

where *d* denotes the decrease in the experimentally determined fraction of infected cells relative to no drug, *D* is the drug concentration, and *IC*_*50*_, and *h* are the open parameters for the fit [89]. The number of infectious viruses per target cell (*m*) delivered in coculture infection was determined by fitting

$$Tx=\;\frac{I_{drug}}{I}=(1-e^{-md})/(1-e^{-m}),$$ (4)

where *Tx* is the experimentally determined number of coculture infected cells in the presence of different RAL concentrations normalized by the number of infected cells in the absence of RAL [27], and *d* is determined for each drug concentration by Eq 3. Script is provided (S2 Script).

## Supporting Information

S1 Fig Image analysis strategy. The mCherry fluorescent signal was thresholded to a binary mask and the number of circular objects in the image was detected using the Matlab Image Analysis Toolbox. From top to bottom, in processing order: 1) all cells, phase contrast; 2) mCherry signal from target cells; 3) binary thresholded mCherry signal; 4) mCherry cell centers (kernels); 5) phase, mCherry signal, and kernel overlay. Bar is 15μM. GFP and CTFR signals underwent the same binary thresholding. The number of mCherry positive 16 pixel^2^ squares around the cell centers, negative for fluorescence in the CTFR channel and positive for fluorescence in the GFP channel, was used as the number of infected target cells.
(TIF)

S2 Fig Fusion exclusion is necessary to accurately quantify infection at early time-points. Lack of fusion exclusion results in a baseline of coculture infection at the earliest time points. Data as in Fig 1C, except CTFR was not used to exclude donor-target fusion events.
(TIF)

S3 Fig Inhibition of additional infection cycles using ATV in cell lines and primary cells. Data is from coculture infections, and transmission index (Tx) is calculated as the number of target cells infected in the presence of ATV divided by the number of target cells infected in the absence of ATV. (**A**) RevCEM clones. (**B**) MT-4 cells. (**C**) PBMCs. Shown are means and standard errors of duplicates. One of three independent experiments for each cell type.
(TIF)

S4 Fig Raw percent of infected target cells in coculture and cell-free infection. Data as in Fig 1C, except no normalization was applied.
(TIF)

S5 Fig NL-AD8 infected donor PBMCs infect PBMCs but are unable to infect G2 targets. Left two bars show infection of PBMCs by PBMC donors infected with NL-AD8 (red) or NL4-3 (blue). Right two bars show the percent of G2 infected after coculture with the same number of PBMC donors infected with either NL-AD8 or NL4-3. Shown are means and standard errors of duplicates. One of three independent experiments.
(TIF)

S6 Fig Gating strategy to detect CFP, YFP, and CFP/YFP co-infected primary CD4^+^ T cells. Percent infected cells shown for CFP (top left quadrant), YFP (bottom right quadrant), and CFP/YFP co-infected (top right).
(TIF)

S7 Fig Gating strategy to detect infected target cell frequency in primary CD4^+^ T cell infection. Donors were labelled with CFSE and infection was assayed by flow cytometry following p24 staining for HIV Gag. Top row is coculture infection, bottom row is cell-free infection. Percent of infected targets in the population (bottom right quadrant) shown in red, and values for other subpopulations in black.
(TIF)

S1 Table Markers for infection. (TIF)

S1 Movie Time-lapse microscopy of RevCEM clone infection. Cells were imaged for GFP, mCherry, and CTFR fluorescence using time-lapse microscopy. Time is hours:minutes post-infection, bar is 20μM. Infected GFP^+^, mCherry^+^ target cells appear as yellow, CTFR^+^ donor cells as blue. ATV was added after wash and before the start of the movie to bracket infection to a 2-hour time window. Hence few new transmissions of viable virus occurred during the movie.
(MP4)

S2 Movie Time-lapse microscopy of MT4 cell infection by cell-free HIV. Cells were imaged for YFP and mCherry, fluorescence using time-lapse microscopy. Time is hours:minutes post-infection, bar is 20μM. Infected YFP^+^, mCherry^+^ cells appear as yellow. ATV was added after wash and before the start of the movie to bracket infection to a 2-hour time window.
(MP4)

S1 Script Global fitting of time-lapse data using Gamma distribution. Python.
(PY) Click here for additional data file.

S2 Script Drug sensitivity model. Matlab.
(M) Click here for additional data file.
