# Supplementary figures and images for: HIV Cell-to-Cell Spread Results in Earlier Onset of Viral Gene Expression by Multiple Infections per Cell
Source: PLoS Pathog. 2016 Nov 3;12(11):e1005964. doi: 10.1371/journal.ppat.1005964 (PMC5094736; doi:10.1371/journal.ppat.1005964)

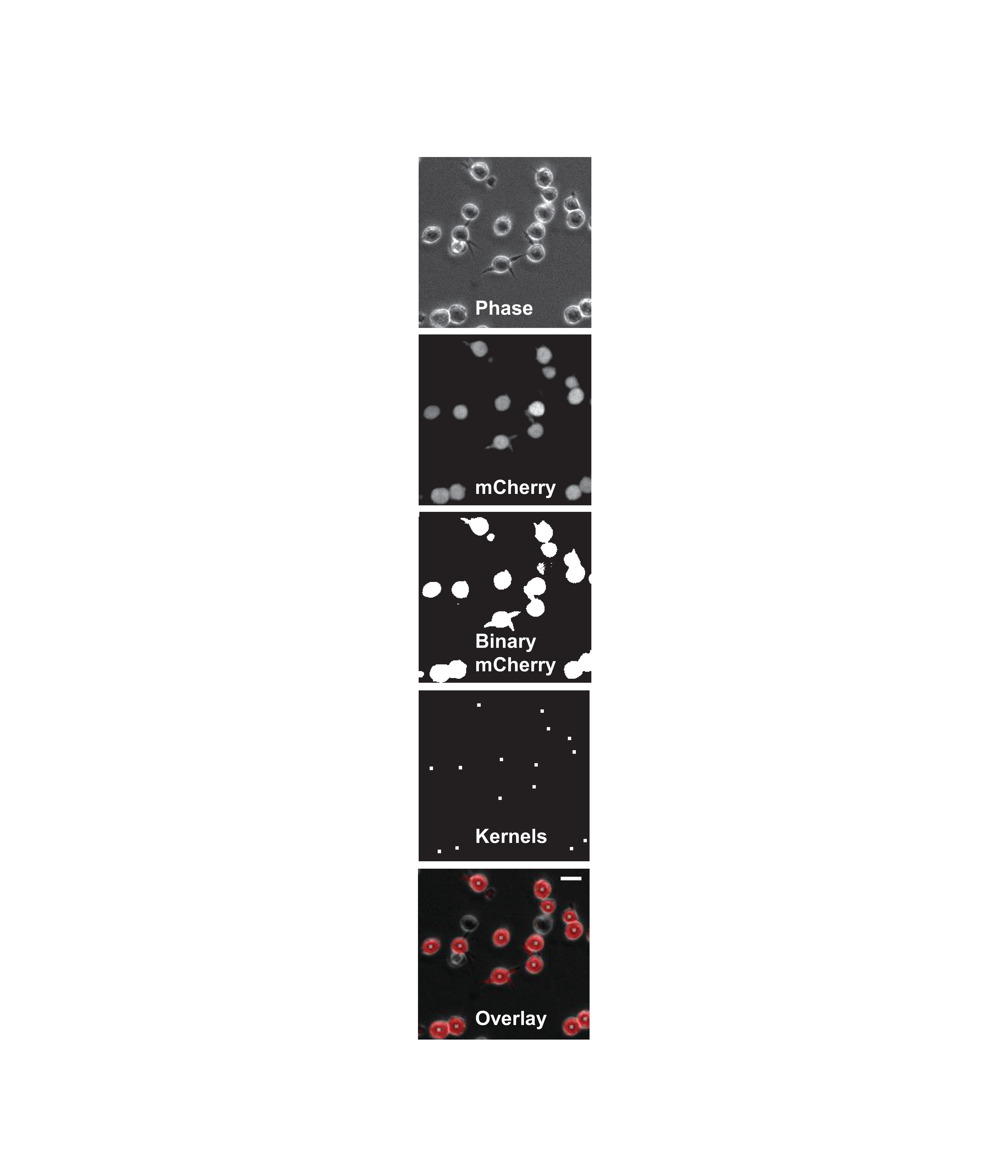

Supplement: S1 Fig — The mCherry fluorescent signal was thresholded to a binary mask and the number of circular objects in the image was detected using the Matlab Image Analysis Toolbox. From top to bottom, in processing order: 1) all cells, phase contrast; 2) mCherry signal from target cells; 3) binary thresholded mCherry signal; 4) mCherry cell centers (kernels); 5) phase, mCherry signal, and kernel overlay. Bar is 15μM. GFP and CTFR signals underwent the same binary thresholding. The number of mCherry positive 16 pixel2 squares around the cell centers, negative for fluorescence in the CTFR channel and positive for fluorescence in the GFP channel, was used as the number of infected target cells. (TIF) [file ppat.1005964.s001.tif]

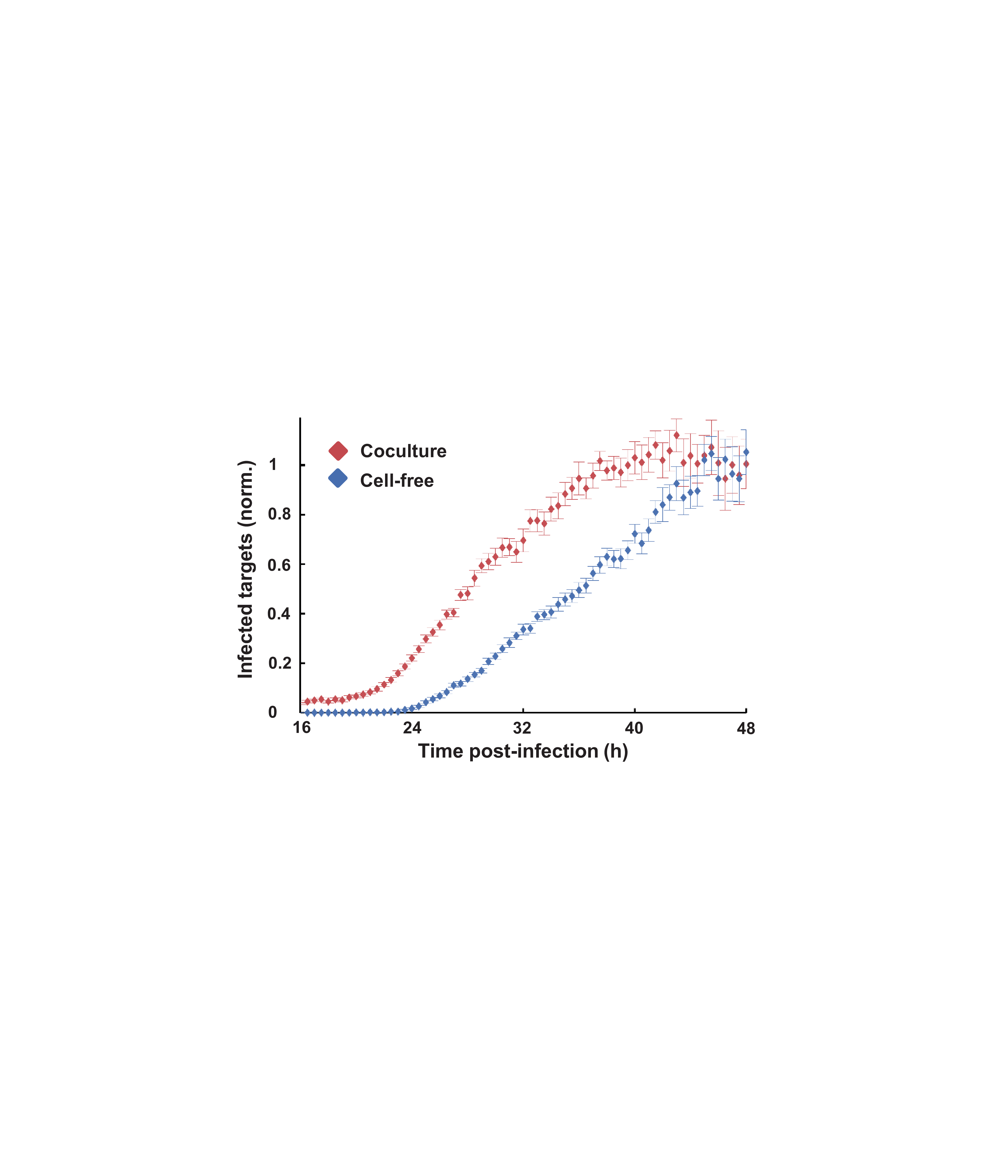

Supplement: S2 Fig — Lack of fusion exclusion results in a baseline of coculture infection at the earliest time points. Data as in Fig 1C, except CTFR was not used to exclude donor-target fusion events. (TIF) [file ppat.1005964.s002.tif]

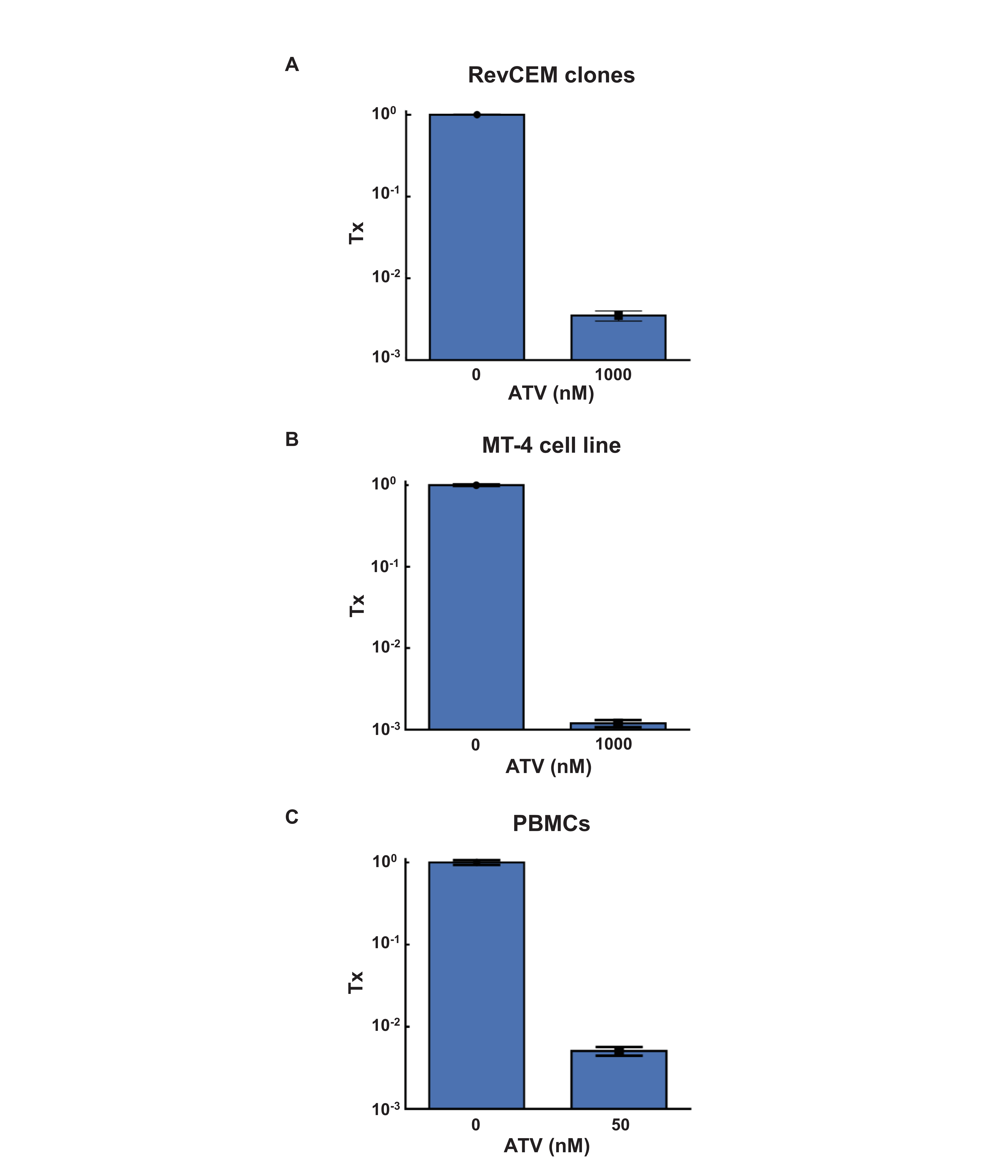

Supplement: S3 Fig — Data is from coculture infections, and transmission index (Tx) is calculated as the number of target cells infected in the presence of ATV divided by the number of target cells infected in the absence of ATV. (A) RevCEM clones. (B) MT-4 cells. (C) PBMCs. Shown are means and standard errors of duplicates. One of three independent experiments for each cell type. (TIF) [file ppat.1005964.s003.tif]

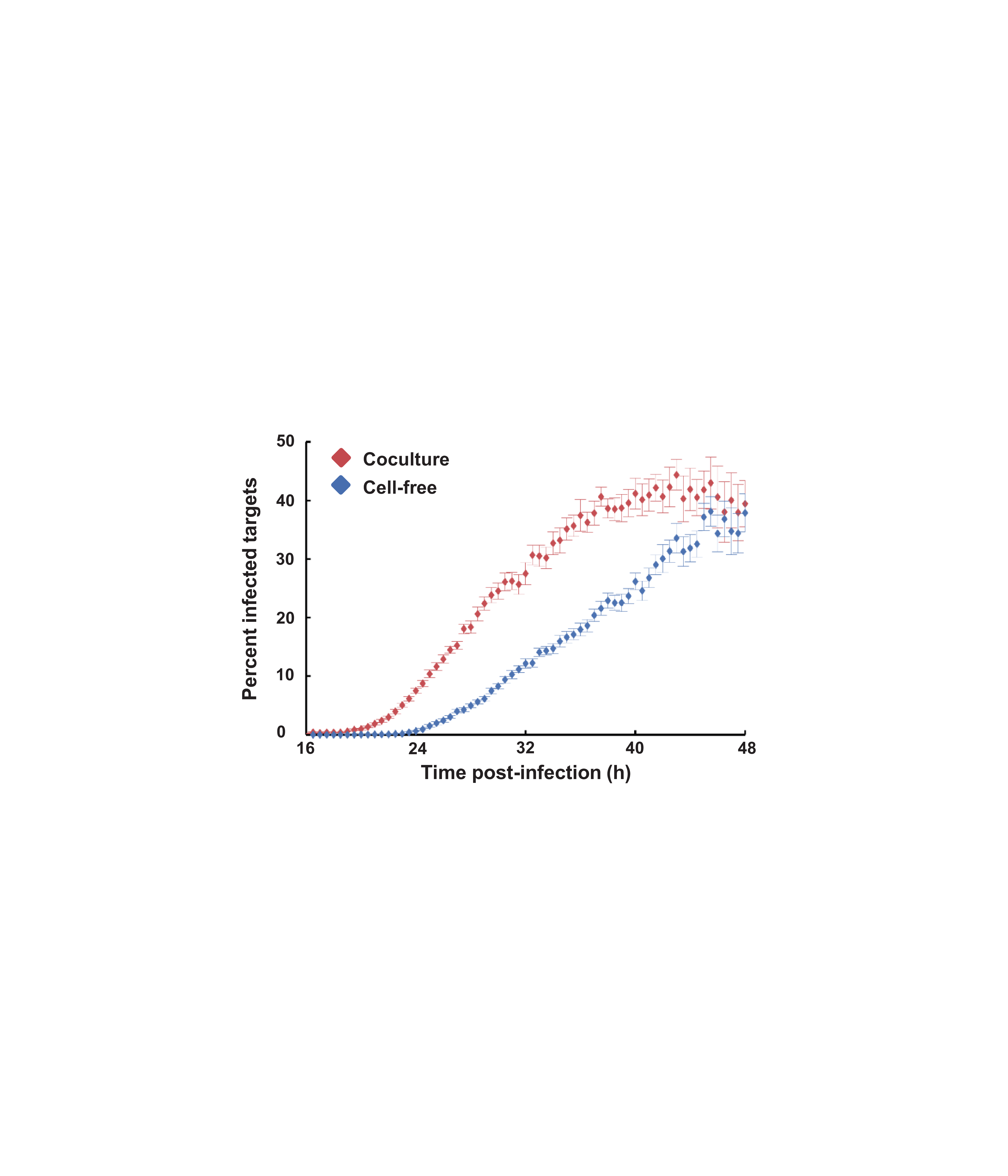

Supplement: S4 Fig — Data as in Fig 1C, except no normalization was applied. (TIF) [file ppat.1005964.s004.tif]

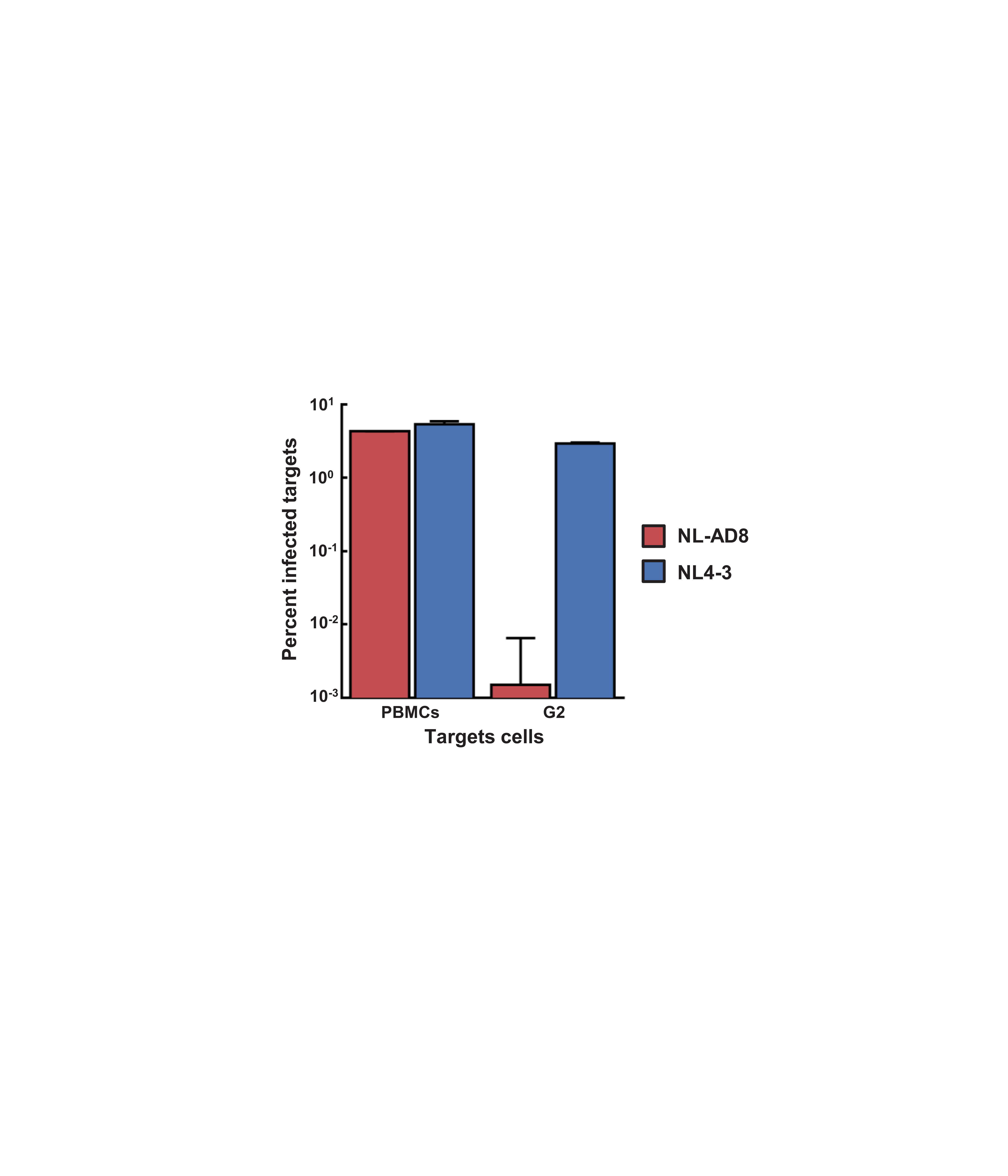

Supplement: S5 Fig — Left two bars show infection of PBMCs by PBMC donors infected with NL-AD8 (red) or NL4-3 (blue). Right two bars show the percent of G2 infected after coculture with the same number of PBMC donors infected with either NL-AD8 or NL4-3. Shown are means and standard errors of duplicates. One of three independent experiments. (TIF) [file ppat.1005964.s005.tif]

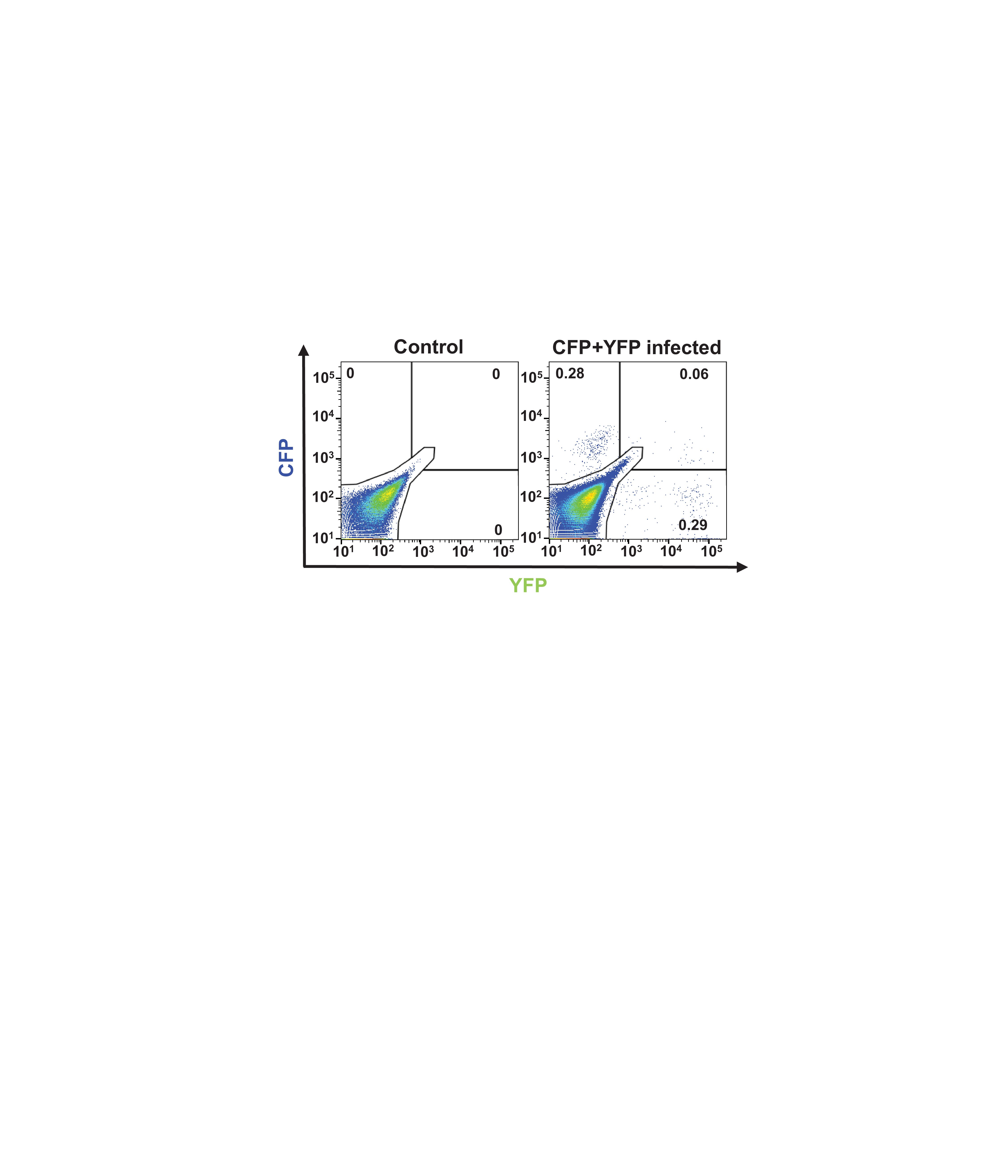

Supplement: S6 Fig — Percent infected cells shown for CFP (top left quadrant), YFP (bottom right quadrant), and CFP/YFP co-infected (top right). (TIF) [file ppat.1005964.s006.tif]

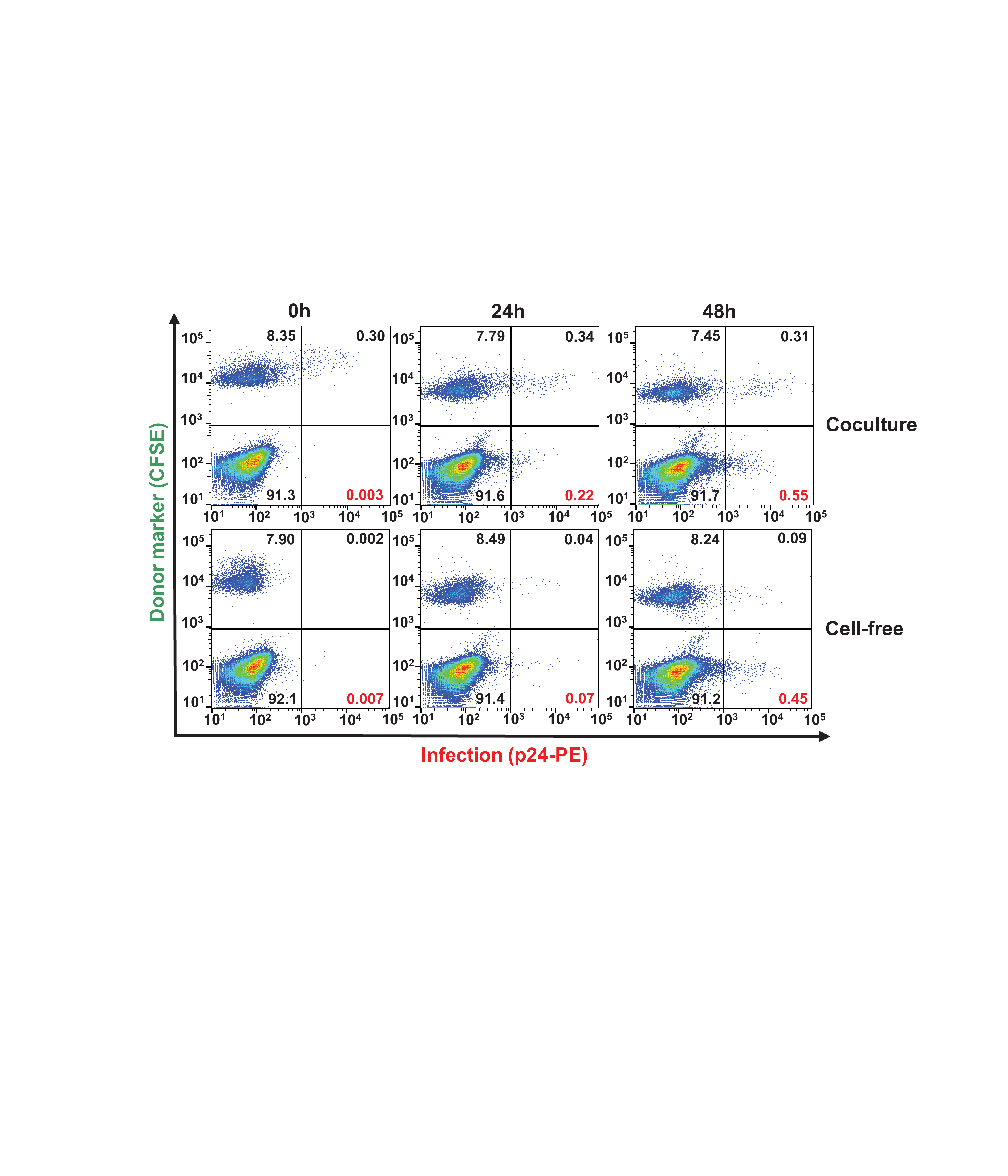

Supplement: S7 Fig — Donors were labelled with CFSE and infection was assayed by flow cytometry following p24 staining for HIV Gag. Top row is coculture infection, bottom row is cell-free infection. Percent of infected targets in the population (bottom right quadrant) shown in red, and values for other subpopulations in black. (TIF) [file ppat.1005964.s007.tif]

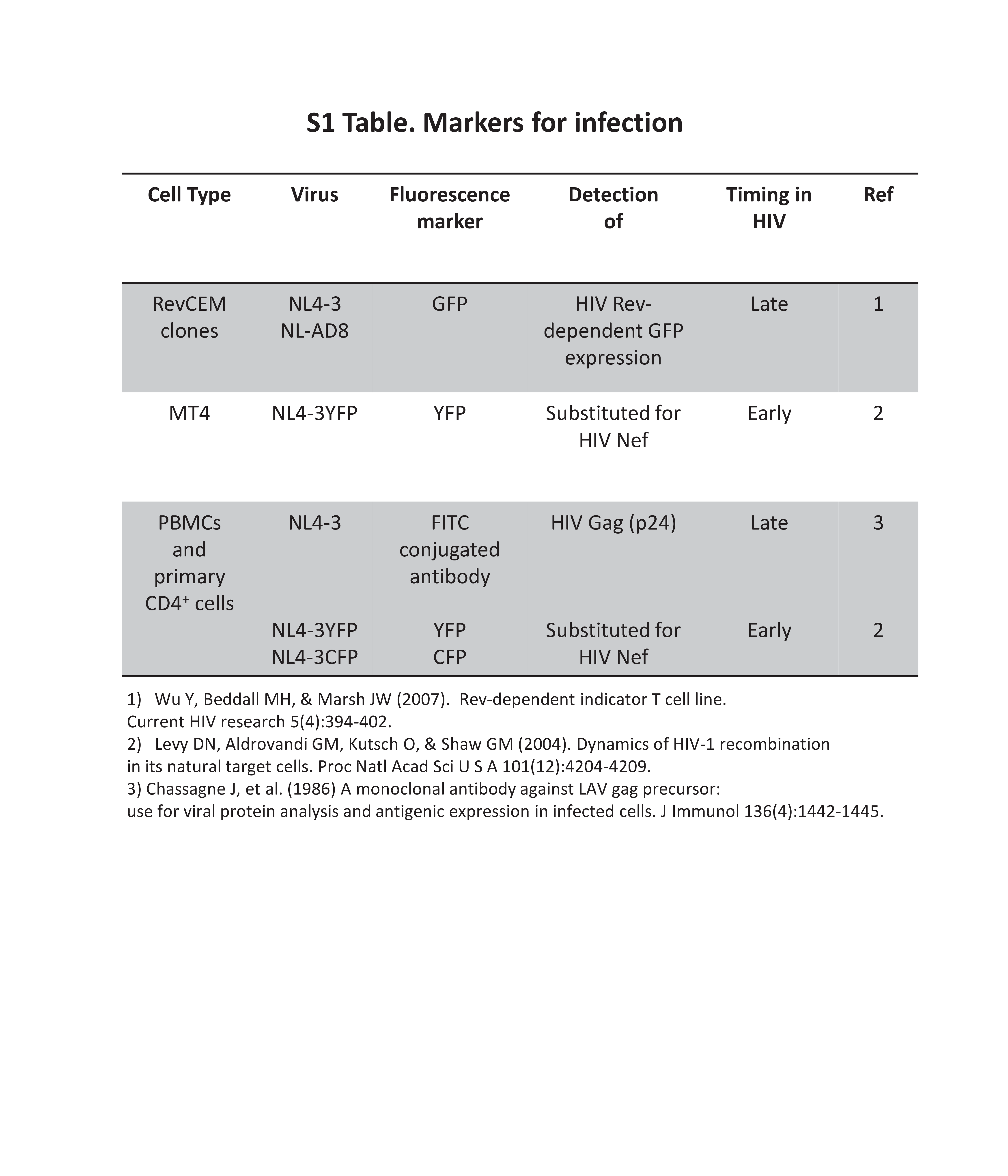

Supplement: S1 Table — (TIF) [file ppat.1005964.s008.tif]
